# Effects of Microgravity, Hypergravity, and Ionizing Radiation on the Enzymatic Activity of Proteinase K

**DOI:** 10.3390/molecules31020229

**Published:** 2026-01-09

**Authors:** Bartosz Rybacki, Wojciech Wysocki, Tomasz Zajkowski, Robert Brodzik, Beata Krawczyk

**Affiliations:** 1 Department of Biotechnology and Microbiology, Faculty of Chemistry, Gdansk University of Technology, 80-233 Gdansk, Poland; 2 Research and Development Department, QIAGEN Gdansk (Blirt SA), 80-172 Gdansk, Poland; robert.brodzik@qiagen.com; 3 Polish Astrobiology Society, 02-697 Warsaw, Poland; wojciechtadwysocki@gmail.com (W.W.); tzajkowski@agh.edu.pl (T.Z.); 4 Faculty of Mechanical Engineering and Ship Technology, Gdansk University of Technology, 80-233 Gdansk, Poland; 5 Space Technology Centre, AGH University of Krakow, 30-059 Krakow, Poland; 6 Blue Marble Space Institute of Science, 600 1st Avenue, Seattle, WA 98104, USA

**Keywords:** proteinase K, enzymatic activity, microgravity, hypergravity, gamma radiation, space biology, molecular biotechnology

## Abstract

Space conditions offer new insights into fundamental biological and molecular mechanisms. The study aimed to evaluate the enzymatic activity of proteinase K (PK) under extreme conditions relevant to space environments: simulated microgravity, hypergravity, and gamma radiation. PK activity was tested using azocasein (AZO) as a chromogenic substrate, with enzymatic reactions monitored spectrophotometrically at 450 nm. A rotating wall vessel (RWV) simulated microgravity, centrifugation at 1000× *g* (3303 rpm) generated hypergravity, and gamma radiation exposure used cesium-137 as the ionizing source. PK activity showed no remarkable changes under microgravity after 16 or 48 h; however, higher absorbance values after 96 h indicated enhanced AZO proteolysis compared to 1 g (Earth gravity) controls. In hypergravity, low PK concentrations exhibited slightly increased activity, while higher concentrations led to reduced activity. Meanwhile, gamma radiation caused a dose-dependent decline in PK activity; samples exposed to deep-space equivalent doses showed reduced substrate degradation. PK retained enzymatic activity under all tested conditions, though the type and duration of stress modulated its efficiency. The results suggest that enzyme-based systems may remain functional during space missions and, in some cases, exhibit enhanced activity. Nevertheless, their behavior must be evaluated in a context-dependent manner. These findings may be significant to advance biotechnology, diagnostics, and the development of enzyme systems for space applications.

## 1. Introduction

Understanding the behavior and mechanisms of bioorganic macromolecules is fundamental to both basic and applied life sciences. Exposing such molecules to unconventional or extreme environmental conditions within a reaction system provides opportunities to analyze and understand mechanisms previously disregarded or overlooked.

Extreme conditions are physical or chemical environmental factors that fall outside of the typical range required for the proper functioning of living organisms and can adversely affect their structure and function. These include temperature extremes, altered pH, osmolarity, high or low pressure, radiation, and the presence of various chemical agents. The current definition of extreme environments is anthropocentric and includes any environment within which key physicochemical parameters deviate from the human-centered norm [1]. Such changes in environmental conditions can significantly impact the structure and activity of bio-organic systems.

Estimates indicate that life began to develop on Earth around 3.9 billion years ago and that humans have been on Earth for 200,000 years. Gravity, although not always tangible or detectable, is an ever-present force that has charted the course of life’s evolutionary adaptation on Earth. Since gravity points directly toward the center of Earth, it determines the form and shape of all objects, including living organisms and bioorganic molecules like structural or enzymatic proteins [2]. Therefore, if gravity influences the origins of enzymatic reactions, would altering this fundamental and transcendent evolutionary factor alter the reaction mechanisms of key biomolecules, such as enzymes?

Any biological object on the Earth’s surface is affected by an average Earth acceleration of 9.81 m/s^2^ (1 g). Microgravity, a state of apparent weightlessness or zero gravity is a term often used to describe a system in which gravitational forces are reduced to seemingly negligible magnitudes [3] (Griffith & Goka, 2023). Although the term is theoretical, because gravity is a force with an infinite range between bodies, microgravity is defined as a fractional value of gravity between 10^−2^ and 10^−6^ g [4], which can have distinct effects on animal physiology and on microorganisms. Indeed, a study on *Escherichia coli* showed that 16 different mutations occurred in the bacterial genome under simulated microgravity conditions, resulting in 60–75% faster growth of bacterial colonies than under natural conditions [5] (ISS National Lab, 2017).

Under environmental stress, bacterial strains can mutate in numerous ways. Research conducted on the International Space Station (ISS) in 2018 revealed that the bacteria discovered are genetically and functionally different from those living on Earth [5]. Microgravity during spaceflight and modeled microgravity analogs (MMA) alter the gene expression and physiology of pathogens [6]. Human bacterial pathogens have been shown to exhibit increased virulence, antibiotic resistance, stress tolerance, and reduced LD50 in animal hosts. For example, the foodborne pathogens *Salmonella enterica* and *E. coli* evaded and suppressed plant innate immunity to colonize intracellular spaces, emphatically illustrating a significant correlation between microgravity and the behavior of animate systems. Our research aims to advance understanding of enzymatic systems, specifically the proteinase K (PK)-azocasein (AZO) model, under microgravity using a rotating wall vessel (RWV) to simulate microgravity conditions experimentally.

The effect of simulated hypergravity on living organisms relies on the sedimentation produced during centrifugation. A centrifuge uses a direct current (DC) motor drive, with the object of investigation located around its vertical axis, causing separation of denser, heavier substances based on their size, viscosity, and rotor speed. The magnitude of gravity is determined by the distance of a test object from the axis of rotation, the angular velocity, and the rotational speed, with the object experiencing centrifugal acceleration [7]. For example, a European Space Agency centrifuge was used to stimulate C2C12 cells at stimulated hypergravity intensities of 5 g, 10 g, and 20 g for two h. The tests indicated that hypergravity positively affects myoblast proliferation and differentiation [7]. The current study investigated the effects of simulated microgravity on the PK-AZO system using representative environments with varying gravity values.

Another key factor of extreme environments that we analyzed in our study is radiation. The generally accepted division of radiation depends on energy, source, and composition, and it can be further categorized as ionizing (i.e., causing ionization of a material medium, such as the detachment of at least one electron of an atom) and non-ionizing [8]. For living organisms, sources of radiation include ultraviolet (UV) radiation, X-rays, gamma radiation, and cosmic rays. All types of ionizing radiation, especially gamma and UV, act on organisms through direct and indirect mechanisms (e.g., via reactive oxygen species). Most often, it is the reactive oxygen species formed by the effects of ionizing radiation that lead to damage to the structures of lipids, carbohydrates, proteins, and nucleic acids [9].

The purpose of the following work was to investigate how simulated microgravity, hypergravity, and gamma radiation affect the enzymatic activity of PK, whether PK retains its enzymatic activity under these extreme conditions, and to assess the potential implications of these interactions for the development of biotechnology and medical diagnostics.

PK is a broad-spectrum serine protease that breaks the peptide bond adjacent to the carboxyl group of an aliphatic and aromatic amino acid, along with a blocked alpha-amino group [10]. Classified as a subtilisin-like protease, a group of serine proteases of 18 to 90 kDa produced by yeast via extracellular secretion, PK can degrade many proteins, such as during regulatory processes of the immune system or in molecular biology [11], and is characterized by relatively high activity, stability, and substrate specificity [12,13]. Due to its wide range of applications, its importance in the industrial market, and the potential benefits of microgravity effects, this enzyme was chosen for studies of enzymatic activity under extreme conditions. AZO was selected to investigate PK proteolytic activity due to its well-documented ability to yield consistent, quantifiable results, making it a reliable substrate for assessing enzyme activity under various experimental conditions [14].

PK plays an essential biological role and is widely used in biotechnology research and industry [15,16,17]. Nevertheless, to our knowledge, it has not yet been investigated under extreme space-related conditions. Here, we examined its enzymatic activity under simulated microgravity, hypergravity, and gamma radiation.

## 2. Results

### 2.1. Effect of Microgravity on the Enzymatic Activity of Proteinase K in Reaction with Azocasein

**Figure 1:**
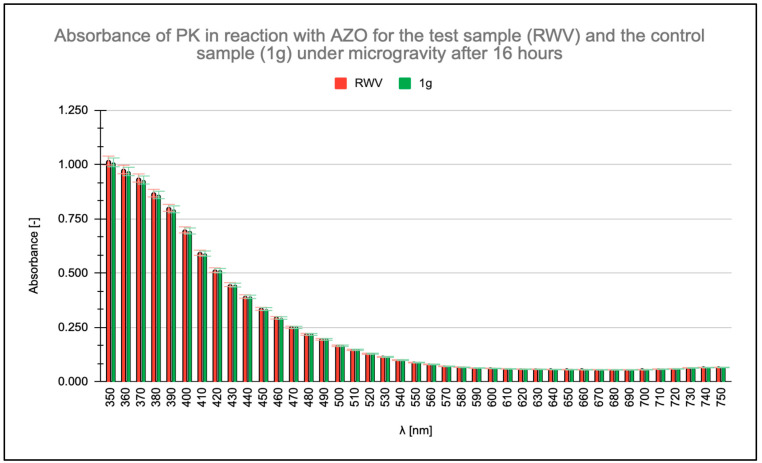
Measured absorbance results for test (rotating wall vessel [RWV]) and control (1 g) samples when studying the enzymatic activity of proteinase K (PK) in reaction with azocasein (AZO) after 16 h. Samples labeled RWV were subjected to continuous microgravity. Trials marked K denote control trials that were also on RWV rotating disks but were oriented parallel to the Earth’s plane and thus subjected to Earth’s gravity (1 g). The “K” orientation served as an internal RWV control with a fixed gravity vector while maintaining identical physical parameters. This condition does not represent static 1 g; however, it still serves as a 1 g reference. By orienting the control parallel to the ground, the system was exposed to stronger sedimentation vectors across the vessel’s cross-section.

**Figure 2:**
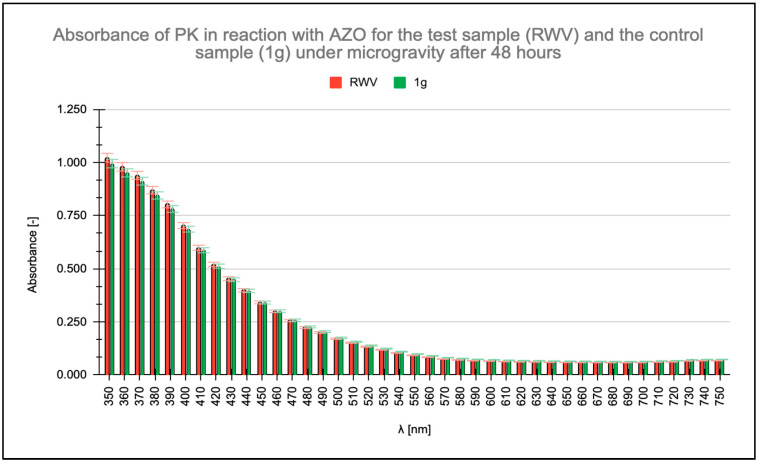
Measured absorbance results for test (rotating wall vessel [RWV]) and control (1 g) samples when studying the enzymatic activity of proteinase K (PK) in reaction with azocasein (AZO) after 48 h.

Absorbance measurements were conducted within the wavelength range of λ = 350 nm to λ = 750 nm, with particular focus on the absorbance maximum of the AZO chromophore group of AZO at λ = 450 nm. Readings were taken at the initiation of the enzymatic reaction and after 16, 48, and 96 h. All measurements were taken for a PK concentration of 0.6 μg/mL. No change in PK activity was observed after 16 h (Figure 1), and no significant differences appeared in the absorbance readings of the ongoing PK-AZO reaction for 48 h (Figure 2). Higher absorbance values were recorded after 96 h of the reaction under simulated microgravity conditions.

During each sampling of the test and control systems for spectrophotometric measurements, syringes were used bidirectionally, i.e., the equivalent volume of the substrate used was replenished and returned to the system. Due to the specificity of the microgravity simulation device (RWV), sample volumes were limited to 200 µL relative to the total 50 mL and immediately reinjected to maintain overall composition and working volume, minimizing impact on reaction molarity and enabling absorbance measurements. Additionally, the collection and reinjection of 200 µL was necessary to prevent air bubble formation, which disrupts microgravity conditions when the vessel is not completely filled. Aliquots were withdrawn solely for real-time monitoring of absorbance, without quenching the reaction at each time point.

**Figure 3:**
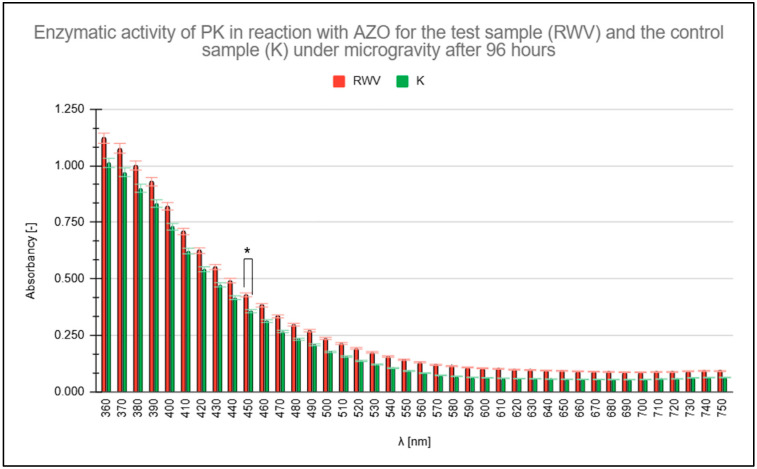
Measured absorbance results for test (rotating wall vessel [RWV]) and control (1 g) samples when studying the enzymatic activity of proteinase K (PK) in reaction with azocasein (AZO) after 96 h. * statistically significant result.

Figure 3 illustrates the predominance of PK activity under simulated microgravity conditions compared to the control (K) conditions of 1 g (Earth gravity). The higher absorbance values for the test sample (RWV), highlighted in red, persist across the analyzed wavelength range.

**Figure 4:**
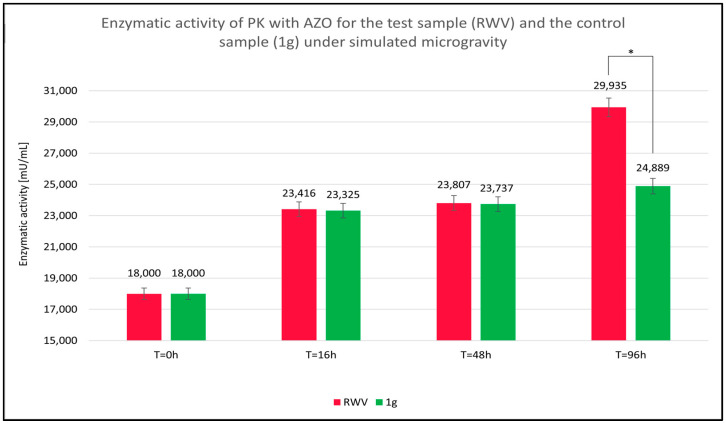
Calculated relative enzymatic activity for test (rotating wall vessel [RWV]) and control (1 g) samples of proteinase K (PK) in reaction with azocasein (AZO) under simulated microgravity after 96 h. * statistically significant result.

The key value is the relative enzymatic activity of the sample (*EA_S_*), calculated as the quotient of the absorbance value of the sample at time T_X_ (*A_Tx_*) and the absorbance of the reference at time T = 0 h (*A_Ref_*), multiplied by the enzymatic activity of the reference (*EA_Ref_*). The relative enzymatic activity values of PK with AZO, shown in Figure 4, were calculated using the following equation:

$${EA}_{S}=\left(\frac{A_{T_{X}}}{A_{Ref}}\right)\times {EA}_{Ref}$$

The summary of the data on the absorbance and relative enzymatic activity for simulated microgravity studies of proteinase K in azocasein is shown Table 1.

Over time, as suggested by the observed trend in absorbance changes, microgravity appears to enhance PK’s ability to degrade AZO. The effect likely reflects a greater release of chromophore groups from the native AZO molecule, resulting in approximately 20% higher values of relative PK (EA_S_) enzymatic activity in the test sample subjected to simulated microgravity (RWV) than in the control sample (1 g).

### 2.2. Effect of Hypergravity on the Enzymatic Activity of Proteinase K in Reaction with Azocasein

**Figure 5:**
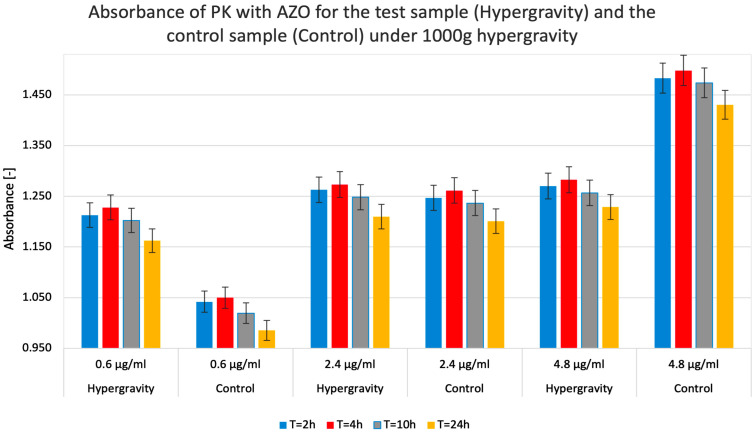
Measured absorbance results for test (Hypergravity) and control (Control) samples when studying the enzymatic activity of proteinase K (PK) in reaction with azocasein (AZO) under hypergravity. The higher the absorbance value, the higher the relative PK activity in a given system. The colors of the columns indicate the test system’s exposure time to hypergravity (1000× *g* centrifugation).

Regardless of the duration of the hypergravity state and the concentrations used, the absorbance ratios for each test system remained consistent. For example, the absorbance values for the 0.6 µg/mL concentration showed a similar ratio to those for the 4.8 µg/mL concentration at all four measurement points (T = 2 h, T = 4 h, T = 10 h, and T = 24 h). This observation speaks to the accuracy of the experiments performed and excludes potential precipitation within the analyzed systems that could disturb further measurements. Figure 5 shows that PK’s ability to degrade the substrate (i.e., activity) depended on its concentration and, to a lesser extent, on the hypergravity exposure time (reaction time).

The summary of the data on the absorbance and relative enzymatic activity for simulated hypergravity studies of proteinase K in azocasein is shown in Table 2.

**Figure 6:**
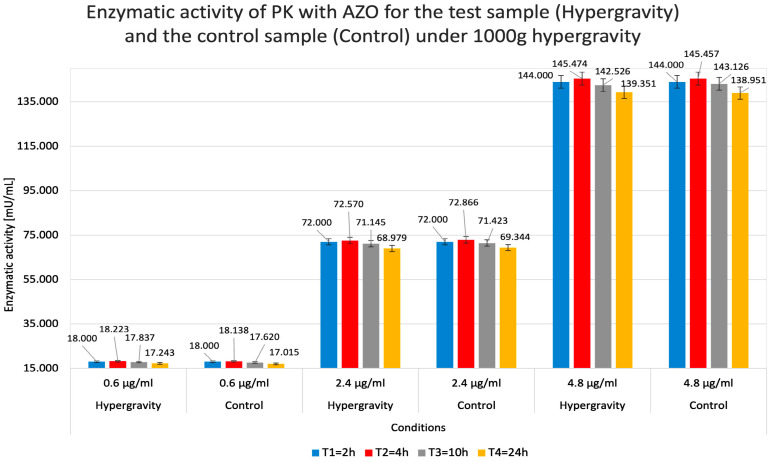
Calculated relative enzymatic activity for test (Hypergravity) and control (Control) samples of proteinase K (PK) in reaction with azocasein (AZO) under simulated hypergravity after 2, 4, 10, and 24 h.

Figure 6 indicates a negligible tendency for lower concentrations of the test substance to produce ‘less visible’ relative activity values than related absorbance measurements. PK relative activity was higher in hypergravity than in control gravity conditions (1 g). In contrast, at higher PK concentrations, hypergravity had a minor negative effect on its activity compared to the control.

### 2.3. Effect of Gamma Radiation on the Enzymatic Activity of Proteinase K in Reaction with Azocasein

**Figure 7:**
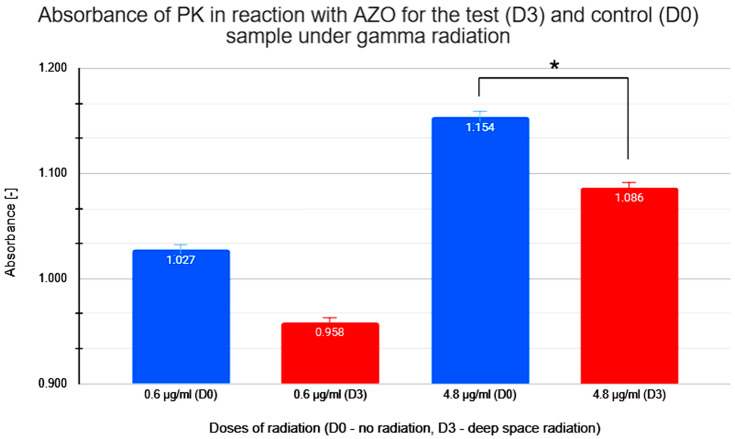
Measured absorbance results for test (D3) and control (D0) samples when studying the enzymatic activity of proteinase K (PK) in reaction with azocasein (AZO) under gamma irradiation. * statistically significant result.

The graph in Figure 7 shows the effect of ionizing radiation from radioactive cesium on PK’s ability to degrade AZO. Samples were subjected to a control dose without radiation (D0) and a test dose equivalent to the radiation level found in deep space of 60 mGy (D3). An experimental series was conducted to test both doses at PK concentrations of 0.6 μg/mL and 4.8 μg/mL.

The absorbance values shown in Figure 7 for sample D3 are noticeably lower than the corresponding concentrations (0.6 μg/mL and 4.8 μg/mL) for samples not exposed to gamma radiation (D0).

The summary of the data on the absorbance and relative enzymatic activity for radiation studies of proteinase K in azocasein is shown Table 3.

**Figure 8:**
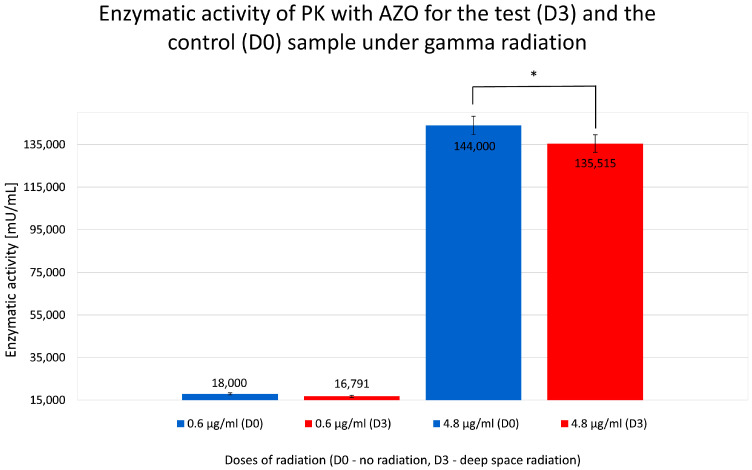
Calculated relative enzymatic activity for test (D3) and control (D0) samples of proteinase K (PK) in reaction with azocasein (AZO) under gamma radiation for D0 (no radiation) and D3 (deep space gamma radiation—60 mGy). * statistically significant result.

However, as expected, exposure to ionizing radiation appeared to slightly inhibit reaction activity (Figure 8), resulting in lower absorbance values, indicating a negative effect of gamma radiation on PK proteolytic activity.

## 3. Discussion

Our experiments demonstrated a positive effect of simulated microgravity on PK proteolytic activity. We suspect that microgravity can potentially increase the spatial availability of interacting molecules, which could act as a catalyst by increasing the contact events between PK molecules and the substrate, AZO. Conventional monolayer assays on solid substrates can support protein or cellular interactions but cannot reproduce the physiological spatial conditions of organisms. Similarly, due to uneven particle distribution and sedimentation, standard liquid media do not reflect naturally occurring fluid dynamics and the spatial accessibility of enzyme substrates under physiological conditions. In contrast, the RWV used in the study to generate simulated microgravity conditions exhibits low fluid shear and reproduces naturally occurring cellular and tissue conditions compared to solid substrates and standard liquid cultures [18]. The RWV levels the centrifugal force vectors and splits the gravity-induced sedimentation velocity into radial and tangential components (Supplementary Materials Figure S1). Through the rotating sidewall of the vessel, the hydrodynamic drag force is directed upward and balances the downward-directed gravitational force [18]. Given that the molecular masses of PK and AZO are similar (28.9 kDa and 23.6 kDa, respectively), it can only be assumed that these particles interact frontally during the reaction. Therefore, we can further only assume that greater spatial availability increases the chance of direct interaction of the two molecules, while the probability of the substrate entering the active site should also increase. RWV generates conditions with reduced sedimentation, meaning that, within a given volume, no forces “pull” the enzyme (or substrate) toward the gravity vector (toward Earth). For this reason, we may presume that the absence of “pull” forces reduces the limiting factor in the reaction between molecules by increasing the availability of the active site and thereby increasing the reaction rate.

Another explanation involves how diffusion operates in weightlessness. Other experiments focused on cerebrospinal fluid reported that the diffusion process is accelerated under microgravity conditions [19], suggesting that the possible reasons for enhanced proteolytic activity may not lie in changes to the enzyme’s structure or functionality, but rather in the differences in the dispersion and penetration of the reaction’s reagents. Biophysical measurements (viscosity, DLS, aggregation analysis, SAXS, etc., as well as enzyme kinetics and Michaelis-Menten models) will be required in future work to test this hypothesis [20,21,22,23,24,25,26,27,28]. Alternatively, a thermodynamic approach to the reaction can also be considered. This argument could suggest that, under microgravity conditions, the enzyme molecule spatially arranges, providing greater energy stability, or that the enzymatic reaction may have a reduced activation energy. Additional studies are required to precisely explain the mechanisms driving the increase in PK’s proteolytic activity with AZO when the reaction happens in an RWV.

As a result of the experiments, hypergravity was found to have variable effects on PK activity. At lower enzyme concentrations, it slightly increases enzyme activity; at higher concentrations, it decreases it. This observation might be explained by the spatial availability of the substrate to the enzyme’s active site. Hypergravity in the test tube system locally restricted the presence of these molecules, which could limit diffusion and contact surface, consequently reducing the efficiency of the enzymatic reaction. This mechanism could be compared to the effect of increased molecular crowding on enzyme activity [29]. At lower enzyme concentrations, hypergravity increased PK activity. We hypothesize that, at lower concentrations, the crowding of enzyme and substrate molecules in a smaller space observed under hypergravity increases the likelihood that the substrate encounters the active site. Therefore, it is likely that the spatial availability of reagents explains the observed effect of hypergravity on PK-mediated AZO degradation. It is noteworthy that between T2 = 4 h and T3 = 10 h, the absorbance values decreased, potentially suggesting PK autolysis. This explanation is supported by the fact that the A [-] readings of the pure substrate did not change [30].

Based on the data presented on the effects of gamma radiation on PK activity, a moderate reduction in the reaction efficiency was observed. Research in this area requires further exploration to formulate conclusive statements, but we suggest that high doses of ionizing radiation can significantly alter the structure and function of enzymes. Studies potentially indicate that ionizing radiation can induce conformational changes in enzymes, altering their active sites and overall structure. For instance, research on chymotrypsin and chymotrypsinogen demonstrated that X-rays can inactivate these enzymes by disrupting their active sites and causing structural damage [31]. Additionally, high-dose ionizing radiation has been found to induce deoxyribonucleic acid (DNA) and ribonucleic acid (RNA) damage, which can further affect enzyme activity and cellular functions [32]. Similarly, high doses of ionizing radiation might disrupt the charge of amino acids in the enzyme’s active site or damage its structure, leading to decreased enzymatic activity. Understanding these effects is crucial for space missions, where exposure to ionizing radiation is inevitable and can have significant implications for biological systems [33,34,35].

These findings indicate that the observed absorbance changes are attributable to enzymatic proteolysis rather than radiation- or microgravity-induced substrate alterations, ensuring that the measured effects reflect true enzymatic activity under the tested conditions.

The outcomes of the current study provide a significant foundation for future, more complex, structural analyses employing CD, SAXS, MS, and/or fluorescence spectroscopy to elucidate the biophysical basis of the observed activity changes. Indeed, studies in this area could include a broader range of enzyme and substrate concentrations, as well as more prolonged exposure to the conditions. The main goal of such analyses would be to determine what specific structural (conformational), spatial, or functional changes (e.g., in the active site) occur under microgravity and increased radiation conditions.

## 4. Materials and Methods

### 4.1. Reagents

PK (20 mg/mL, batch number B160223-F, code RP107-B-B) was obtained from QIAGEN Gdansk (Blirt SA, Gdansk, Poland). Stock activity amounted to 30 U/mg of protein, so activity was 18 mU/mL for concentration A1 (0.6 μg/mL), 72 mU/mL for concentration C2 (2.4 μg/mL), and 144 mU/mL for concentration D1 (4.8 μg/mL).

As the manufacturer states, one unit of PK hydrolyzes urea-denatured hemoglobin to produce a color equivalent to 1 μmol of tyrosine per 1 min at 37 °C and pH 7.5 (Folin & Ciocalteu’s method) [36]; 1 U = 1 mAnsonU. Azocasein (AZO, Cat. Number: A2765-1G, LOT Number: SLCM2806, US) and phosphate-buffered saline (PBS, 1×, Cat. Number: 524650-1EA, LOT Number: 4113386, Burlington, MA, USA) were both purchased from Sigma-Aldrich (St. Louis, MO, USA)/Merck (Darmstadt, Germany). Ultrapure water was prepared using a Milli-Q purification system (Merck Millipore, Darmstadt, Germany).

### 4.2. Proteinase K Activity Methodology

Given the many applications of PK, it is crucial to choose a relatively simple, transparent assay for its activity. It is worth noting that the publication described here unifies concepts of activity and stability. Thus, the activity represents the relative and approximate activity of an enzyme, based on its sustained ability over time to degrade the chromophoric substrate, AZO, resulting in a colorful and spectrophotometrically measurable reaction [14].

PK activity was assessed using a modified Charney and Tomarelli method with AZO as the chromogenic substrate [14]. The enzymatic degradation of AZO results in the release of soluble chromophores, with absorbance measured at 450 nm using UV-Vis spectrophotometers (Epoch [BioTek, Agilent Technologies, Santa Clara, CA, USA], Eppendorf Kinetic [Eppendorf, Hamburg, Germany] and HACH DR3900 [HACH, Loveland, CO, USA]). The following combinations of PK and AZO were prepared: AZO1—2 mg/mL AZO in 1× PBS; AZO2—6 mg/mL AZO in 1× PBS; PK concentrations: A1—0.6 μg/mL; C2—2.4 μg/mL; D1—4.8 μg/mL; and C2*—2.4 μg/mL in AZO2. Absorbance values were recorded at designated time points to monitor enzymatic activity. The higher the absorbance value obtained, the more chromophore groups enter the solution, the more AZO molecules are digested by PK, and consequently, the higher the relative activity. In the test procedure described, it was necessary to account for unusual volumes, instruments, and experiment durations. Therefore, concentrations were selected to maximize the reaction time while still allowing observation of absorbance changes as the PK-AZO reaction progressed.

### 4.3. Simulated Microgravity Experiments

To investigate the effects of simulated microgravity, a reaction mixture containing 0.6 μg of PK per 1 mL of AZO1 solution (2 mg/mL AZO in 1× PBS) was prepared and scaled to a final volume of 50 mL to accommodate the capacity of the RWV (developed by the National Aeronautics and Space Administration [NASA] Ames Research Center [ARC]/Synthecon, Houston, TX, USA; Supplementary Materials Figure S1).

Simulated microgravity conditions of approximately 0.01 g were maintained continuously for 96 h. Absorbance measurements were recorded at 450 nm, corresponding to the maximum absorbance of the AZO chromophore. Due to the requirement for prolonged exposure to microgravity, the enzymatic reaction was extended over time. To optimize the experimental conditions and evaluate enzyme activity kinetics, various PK–AZO concentration pairs were tested, and reaction progress was assessed by time-dependent absorbance changes. To mitigate the impact of potential autolysis of Proteinase K, all experimental conditions were conducted under identical incubation times, temperatures, and handling procedures. Consequently, any autolytic degradation would occur uniformly across conditions and not affect relative comparisons.

The RWV setup included a test system oriented parallel to the ground plane (to simulate microgravity) and a control system oriented perpendicular to the ground (to approximate 1 g conditions and promote sedimentation). All experiments were conducted under controlled laboratory conditions at 23.3 °C and 51% relative humidity.

All samples were incubated under aseptic, closed-system conditions in sealed sterile vessels (microtubes, sealed plates, screw-cap vials). Negative controls with buffer and substrate showed no increase in absorbance after 96 h, confirming no microbial contamination. The RWV used for microgravity simulation was inspected before measurements; no bubbles or signs of evaporation were observed.

### 4.4. Hypergravity Experiments

To comprehensively assess the influence of gravitational forces, the study was extended to include the effects of hypergravity on the enzymatic activity of selected PK–AZO systems. PK and AZO were tested at concentrations corresponding to A1—0.6 μg/mL, C2—2.4 μg/mL, and D1—4.8 μg/mL in AZO1 solution (2 mg/mL AZO in 1× PBS). Samples were subjected to continuous centrifugal force equivalent to 1000× *g* (3303 rpm) using a Sigma Polygen 1-1 SPK centrifuge for 24 h.

Reaction mixtures were placed in sterile, tightly sealed 2 mL high-density polypropylene (HD-PP) tubes to eliminate potential interactions between the enzyme and container material. Background absorbance measurements were performed using water, PBS, and the AZO1 substrate solution prior to the enzymatic assays.

Six independent PK-AZO systems were prepared: three concentrations for the hypergravity-exposed test group and three matched concentrations for the control group. The control samples were maintained under identical temperature and humidity conditions on a rocking platform to ensure continuous mixing, and oriented perpendicular to the ground to simulate 1 g conditions.

Sterile absorbance readings were taken at 1, 4, 10, and 24 h by drawing 200 μL aliquots. After each measurement, the sampled volume was returned to the reaction vessel to preserve the original molar ratios of enzyme and substrate throughout the experiment.

### 4.5. Ionizing Radiation Assay

To investigate the effect of ionizing radiation on enzymatic activity, two PK concentrations were tested (A1 [0.6 μg/mL] and D1 [4.8 μg/mL]), each prepared in AZO1 solution (2 mg/mL AZO in 1× PBS). The experimental system was assembled in a sterile 96-well plate, maintaining constant volume and concentration across all wells.

Irradiation was performed using a radioactive cesium source located at the NASA ARC. The enzyme-substrate mixtures were subjected to a defined exposure period under these conditions. The relevant physical radiation parameters, expressed in mGy (D0—no radiation, D3—60 mGy), were calculated for an exposure period to radioactive cesium that corresponded to a radiation dose of 60 mGy. This was intended to simulate the cumulative dose of ionizing radiation encountered in deep space, as reported by Sihver and Mortazavi (2021) [37]. Due to institutional confidentiality agreements, the other technical details regarding the placement and intensity of the cesium source remain undisclosed, but they do not affect the possible replication of the radiation values used in the study.

All samples were processed under sterile conditions, and absorbance was measured post-irradiation at 450 nm, the maximum absorbance wavelength of the AZO chromophore, to assess proteolytic activity.

### 4.6. Analysis of Experimental Calculations

Studies on the effects of various gravitational forces, i.e., microgravity, Earth gravity, and hypergravity, as well as gamma radiation, are feasibility and/or proof of concept tests and require more extensive statistical analysis. The studies are primarily based on spectrophotometric absorbance measurements focused on the absorbance maximum of the AZO chromophore at λ = 450 nm.

For example, in studies on the effect of simulated microgravity in the subsection ‘Effect of microgravity on the enzymatic activity of proteinase K in reaction with azocasein’, a PK concentration of 0.6 μg/mL was used. The PK stock obtained from QIAGEN Gdansk had an activity of 30 U/mg. Converting the activity to 1 mL of reaction solution, we obtained: 30 × 0.0006 = 0.018 U/mL = 18 mU/mL. The same analogy applies to the other PK concentrations.

All measurements for each experiment were performed in at least three repetitions. The coefficient of variation (CV) for all measurements used was less than 10%. A detailed statistical analysis is provided in Files S1–S3 with Tables S1–S19 (Supplementary Materials).

Simulated microgravity experiments were analyzed using paired Wilcoxon signed-rank tests at individual time points and Friedman repeated-measures analysis across time. While no significant differences were detected at early time points, a significant cumulative effect emerged over time, with pronounced absorbance divergence observed after 96 h of exposure.

Hypergravity experiments were evaluated using Friedman repeated-measures analysis, which revealed a statistically significant time-dependent effect across all tested concentrations (χ^2^ = 18.0, df = 3, *p* = 0.0004), indicating systematic changes in absorbance under prolonged hypergravity exposure.

Radiation experiments were analyzed using paired comparisons between non-irradiated (D0) and irradiated (D3) samples. Due to the limited number of biological replicates, the Wilcoxon signed-rank test was applied as the primary inferential method, with a paired Student’s *t*-test used as a descriptive reference. While radiation-induced absorbance changes did not reach statistical significance at lower protein concentration, a consistent directional decrease was observed. At higher concentration, radiation exposure resulted in a statistically significant reduction in absorbance (*p* < 0.05), confirming the sensitivity of the experimental setup.

## 5. Conclusions

In conclusion, we were able to determine the impact of different gravitational conditions, i.e., microgravity (0.01 g), Earth gravity (1 g), and hypergravity (1000 g), as well as the impact of gamma radiation on the enzymatic activity of PK in reaction with AZO.

Under microgravity conditions, we observed higher relative enzymatic activity of PK in the reaction with AZO than in the control sample (1 g) after 96 h. In hypergravity exposure, low PK concentrations showed slightly increased activity, while higher concentrations led to decreased activity. Gamma radiation caused a dose-dependent decrease in PK activity. Samples exposed to doses corresponding to radiation in deep space showed reduced relative enzymatic activity. PK retains its enzymatic activity under all conditions tested, and the type and duration of stress modulate its effectiveness.

This research may be helpful for designing bioproduction processes in space, for research on international space stations, and for a more accurate understanding of enzyme properties for the molecular biotechnology and biopharmaceutical sectors. The results require more in-depth analysis in the future, while suggesting potential significance for future research on space bioprocesses.

## Figures and Tables

**Table 1:** Summary of absorbance data [-] and relative enzymatic activity for simulated microgravity studies [mU/mL] of proteinase K in azocasein.

Parameter	RWV	1 g
Absorbance T = 0 h	0.258	0.258
Activity T = 0 h	18.000	18.000
Absorbance T = 16 h	0.336	0.334
Activity T = 16 h	23.416	23.325
Absorbance T = 48 h	0.341	0.340
Activity T = 48 h	23.807	23.737
Absorbance T = 96 h	0.429	0.357
Activity T = 96 h	29.935	24.889

**Table 2:** Summary of absorbance data [-] and relative enzymatic activity for hypergravity studies [mU/mL] of proteinase K in azocasein.

Parameter	Hypergravity	Control
PK concentration = 0.6 μg/mL		
Absorbance T = 2 h	1.213	1.042
Activity T = 2 h	18.000	18.000
Absorbance T = 4 h	1.228	1.050
Activity T = 4 h	18.223	18.138
Absorbance T = 10 h	1.202	1.020
Activity T = 10 h	17.837	17.620
Absorbance T = 24 h	1.162	0.985
Activity T = 24 h	17.243	17.015
PK concentration = 2.4 μg/mL		
Absorbance T = 2 h	1.263	1.247
Activity T = 2 h	72.000	72.000
Absorbance T = 4 h	1.273	1.262
Activity T = 4 h	72.570	72.866
Absorbance T = 10 h	1.248	1.237
Activity T = 10 h	71.145	71.423
Absorbance T = 24 h	1.210	1.201
Activity T = 24 h	68.979	69.344
PK concentration = 4.8 μg/mL		
Absorbance T = 2 h	1.270	1.483
Activity T = 2 h	144.000	144.000
Absorbance T = 4 h	1.283	1.498
Activity T = 4 h	145.474	145.457
Absorbance T = 10 h	1.257	1.474
Activity T = 10 h	142.526	143.126
Absorbance T = 24 h	1.229	1.431
Activity T = 24 h	139.351	138.951

**Table 3:** Summary of absorbance data [-] and relative enzymatic activity for radiation studies [mU/mL] of proteinase K (PK) in azocasein for PK concentrations of 0.6 μg/mL and 4.8 μg/mL.

Parameter	0.6 μg/mL	4.8 μg/mL
Absorbance D0	1.027	1.154
Activity D0	18.000	144.000
Absorbance D3	0.958	1.086
Activity D3	16.791	135.515

## Data Availability

Data are contained within the article and Supplementary Materials.

## Supplementary Materials

The following supporting information can be downloaded at: https://www.mdpi.com/article/10.3390/molecules31020229/s1, Figure S1. Operation of Rotating Wall Vessel (RWV) device. Vector velocity diagram of the forces acting on the particle—based on materials from Synthecon [https://www.synthecon.com/technology; accessed on 21 November 2025]. The attached graphics represent the distribution of force vectors acting on the analyzed object (Given the proteolytic properties of proteinase K (PK), its sensitivity, and activity, uniform access to the substrate in each volume is critical to minimize the effect of randomness and study the enzyme’s performance in a spatial arrangement without sedimentation. The graphics were created based on the materials on https://www.synthecon.com/technology. Statistical analysis is included in the Files S1–S3 (paired *t*-tests, Wilcoxon signed-rank test, etc.) in Supplementary Materials. File S1—Statistical analysis details of radiation results (Tables S1–S4). File S2—Statistical analysis of the hypergravity experiment (Tables S5–S12). File S3—Statistical analysis of the simulated microgravity experiment (Tables S13–S19).

## Abbreviations

The following abbreviations are used in this manuscript: Autonomous Modular Biotechnological Experiment on a Rocket (AMBER), Azocasein (AZO), Deoxyribonucleic Acid (DNA), High-Density Polypropylene (HD-PP), National Aeronautics and Space Administration Ames Research Center (NASA ARC), Phosphate-Buffered Saline (PBS), Proteinase K (PK), Ribonucleic Acid (RNA), and Rotating Wall Vessel (RWV).
