# Supplementary material for: Effects of Microgravity, Hypergravity, and Ionizing Radiation on the Enzymatic Activity of Proteinase K
Source: Molecules. 2026 Jan 9;31(2):229. doi: 10.3390/molecules31020229 (PMC12843933; doi:10.3390/molecules31020229)
Supplement: Supplementary file 1 [file molecules-31-00229-s001.zip › molecules-4045214-supplementary.pdf]

Supplementary Materials

Effects of microgravity, hypergravity, and ionizing radiation on the enzymatic activity of Proteinase K

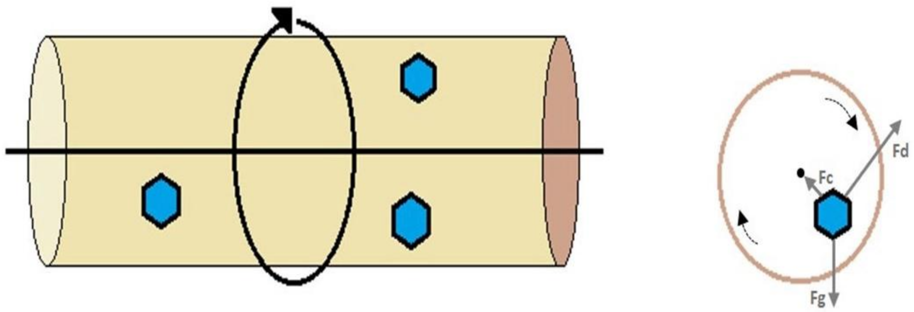

Figure S1. Operation of Rotating Wall Vessel (RWV) device. Vector velocity diagram of the forces acting on the particle. The graphics were created based on the materials on https://www.synthecon.com/technology. Accessed on 21th November 2025.

The attached graphics represent the distribution of force vectors acting on the analyzed object. Given the proteolytic properties of proteinase K (PK), its sensitivity, and activity, uniform access to the substrate in each volume is critical to minimize the effect of randomness and study the enzyme's performance in a spatial arrangement without sedimentation.

File S1 Statistical analysis details of radiation results

Experimental design and statistical rationale

Absorbance measurements were performed for paired samples before (D0) and after gamma radiation exposure (D3) for two protein concentrations (0.6 µg/mL and 4.8 µg/mL). Each sample measured after radiation corresponded directly to its non-irradiated counterpart, forming a paired experimental design.

Given the small sample size (n = 6 per concentration), two complementary statistical approaches were applied:

- A paired Student's t-test was used as a descriptive parametric reference.
- A Wilcoxon signed-rank test was applied as the primary inferential test, as it does not rely on normality assumptions and is appropriate for small-sample feasibility studies.

Table S1. Raw absorbance values before (D0) and after radiation exposure (D3)

0.6 µg/mL		
**Sample**	**D0 (no radiation)**	**D3 (radiation)**
1	0.8698	0.8558
2	1.1042	1.1043
3	1.1076	0.9153
4	1.0526	1.0082
5	0.9882	0.9074
6	1.0379	0.9595
4.8 µg/mL		
**Sample**	**D0 (no radiation)**	**D3 (radiation)**
1	1.1546	1.1201
2	1.1652	1.1185
3	1.1412	1.0196
4	1.1492	1.1029
5	1.1547	1.10524
6	1.1564	1.1021

Wilcoxon signed-rank test calculations

Table S2. Ranked absolute differences (Wilcoxon signed-rank test) for 0.6 μg/mL

0.6 μg/mL				
**Sample**	**ΔA**	**|ΔA|**	**Rank**	**Signed rank**
2	+0.0001	0.0001	1	+1
1	-0.0140	0.0140	2	-2
4	-0.0444	0.0444	3	-3
6	-0.0784	0.0784	4	-4
5	-0.0808	0.0808	5	-5
3	-0.1923	0.1923	6	-6

- Sum of positive ranks: $W^{+} = 1$
- Sum of negative ranks: $W^{-} = 20$
- Test statistic: W=1
- Two-tailed p-value (exact): p≈0.094

Table S3. Ranked absolute differences (Wilcoxon signed-rank test) for 4.8 μg/mL

4.8 μg/mL				
**Sample**	**ΔA**	**|ΔA|**	**Rank**	**Signed rank**
1	-0.0345	0.0345	1	-1
4	-0.0463	0.0463	2	-2
2	-0.0467	0.0467	3	-3
5	-0.0495	0.0495	4	-4
6	-0.0543	0.0543	5	-5
3	-0.1216	0.1216	6	-6

- Sum of positive ranks: $W^{+} = 0$
- Sum of negative ranks: $W^{-} = 21$
- Test statistic: $W = 0$
- Two-tailed p-value (exact): $p \approx 0.031$

Paired Student's t-test (descriptive reference)

Table S4 Paired Student's t-test

Concentration	Mean ΔA	SD	t	df	p-value
0.6 μg/mL	-0.068	0.071	-2.36	5	0.065
4.8 μg/mL	-0.059	0.030	-4.78	5	0.005

Interpretation within the feasibility study framework

For the 0.6 μg/mL concentration, neither the Wilcoxon signed-rank test nor the paired t-test reached statistical significance ($p > 0.05$), although five of six paired differences showed a consistent decrease in absorbance after radiation exposure. This directional consistency suggests a non-random trend detectable by the experimental setup.

For the 4.8 μg/mL concentration, both the Wilcoxon signed-rank test ($p \approx 0.031$) and the paired t-test ($p = 0.005$) indicated a statistically significant decrease in absorbance following radiation exposure. All paired samples exhibited changes in the same direction, supporting a systematic radiation-associated effect.

Within the context of a feasibility study, these results demonstrate that the applied experimental protocol is capable of detecting radiation-induced changes in protein absorbance and provides quantitative estimates of effect size and variability necessary for the design and powering of future confirmatory experiments.

File S2 Statistical analysis of the hypergravity experiment

This File S2 describes the statistical methodology used to evaluate the effect of hypergravity on proteinase K absorbance.

Experimental design

For each tested protein concentration (0.6, 2.4, and 4.8 µg/mL), absorbance was measured for six independent samples (biological replicates). Each sample was observed repeatedly over time under two conditions:

- hypergravity exposure,
- 1g control.

Measurements were performed at the following time points:

- T0: baseline (before hypergravity exposure),
- T1: 2 h,
- T2: 4 h,
- T3: 10 h,
- T4: 24 h.

Thus, the experiment follows a repeated-measures design, with the same independent samples tracked over time.

Table S5. Raw absorbance values under hypergravity conditions

Time	Concentration	Sample 1	Sample 2	Sample 3	Sample 4	Sample 5	Sample 6	Mean
2 h	0.6 µg/mL	1.218	1.206	1.214	1.212	1.210	1.216	1.213
	2.4 µg/mL	1.256	1.264	1.269	1.259	1.263	1.272	1.263
	4.8 µg/mL	1.270	1.267	1.274	1.268	1.272	1.270	1.270
4 h	0.6 µg/mL	1.229	1.222	1.228	1.224	1.227	1.225	1.226
	2.4 µg/mL	1.277	1.269	1.274	1.276	1.272	1.275	1.273
	4.8 µg/mL	1.277	1.280	1.285	1.282	1.278	1.281	1.281
10 h	0.6 µg/mL	1.197	1.201	1.209	1.208	1.202	1.205	1.202
	2.4 µg/mL	1.249	1.247	1.242	1.243	1.250	1.248	1.246
	4.8 µg/mL	1.259	1.258	1.254	1.256	1.259	1.253	1.257
24 h	0.6 µg/mL	1.165	1.161	1.161	1.163	1.160	1.159	1.162

	2.4 µg/mL	1.208	1.205	1.217	1.212	1.209	1.210	1.210
	4.8 µg/mL	1.235	1.223	1.228	1.233	1.229	1.228	1.229

Table S6. Raw absorbance values under control (1g) conditions

Time	Concentration	Sample 1	Sample 2	Sample 3	Sample 4	Sample 5	Sample 6	Mean
2 h	0.6 µg/mL	1.034	1.050	1.042	1.040	1.045	1.043	1.042
	2.4 µg/mL	1.246	1.248	1.247	1.245	1.249	1.248	1.247
	4.8 µg/mL	1.473	1.488	1.488	1.481	1.478	1.487	1.483
4 h	0.6 µg/mL	1.061	1.048	1.041	1.054	1.049	1.052	1.050
	2.4 µg/mL	1.255	1.262	1.268	1.258	1.263	1.260	1.262
	4.8 µg/mL	1.495	1.495	1.502	1.501	1.497	1.500	1.497
10 h	0.6 µg/mL	1.025	1.011	1.025	1.022	1.019	1.017	1.020
	2.4 µg/mL	1.239	1.239	1.232	1.238	1.233	1.236	1.237
	4.8 µg/mL	1.478	1.477	1.464	1.472	1.479	1.470	1.473
24 h	0.6 µg/mL	0.986	0.982	0.988	0,989	0,985	0,981	0.985
	2.4 µg/mL	1.198	1.201	1.204	1,203	1,200	1,199	1.201
	4.8 µg/mL	1.433	1.436	1.423	1,424	1,432	1,435	1.431

Effect definition and preprocessing

To isolate the effect of hypergravity from baseline variability, the hypergravity-induced change in absorbance was defined as:

$$\Delta A(t) \quad = A_{hypergravity}(t) - A_{control}(t)$$

This difference-based metric reduces between-sample variability and is recommended for exploratory repeated-measures designs.

Baseline measurements (T0) were used exclusively for effect detection relative to the pre-exposure state and were not included in the time-dependence analysis.

Table S7. Mean absorbance differences (ΔA)

Concentration	Sample	2 h	4 h	10 h	24 h
0.6 µg/mL	1	+0.184	+0.168	+0.172	+0.179
0.6 µg/mL	2	+0.156	+0.174	+0.190	+0.179
0.6 µg/mL	3	+0.172	+1.187	+0.184	+0.173
0.6 µg/mL	4	+0.172	+0.170	+0.186	+0.174
0.6 µg/mL	5	+0.165	+0.178	+0.183	+0.175
0.6 µg/mL	6	+0.173	+0.173	+0.188	+0.178
2.4 µg/mL	1	+0.010	+0.022	+0.010	+0.010
2.4 µg/mL	2	+0.016	+0.007	+0.008	+0.004
2.4 µg/mL	3	+0.022	+0.006	+0.010	+0.013
2.4 µg/mL	4	+0.014	+0.018	+0.005	+0.009
2.4 µg/mL	5	+0.014	+0.009	+0.017	+0.009
2.4 µg/mL	6	+0.017	+0.015	+0.012	+0.011
4.8 µg/mL	1	-0.203	-0.218	-0.219	-0.198
4.8 µg/mL	2	-0.221	-0.215	-0.219	-0.213
4.8 µg/mL	3	-0.214	-0.217	-0.210	-0.195
4.8 µg/mL	4	-0.213	-0.219	-0.216	-0.191
4.8 µg/mL	5	-0.206	-0.219	-0.220	-0.203
4.8 µg/mL	6	-0.217	-0.219	-0.217	-0.207

Descriptive statistics

For each protein concentration and time point, the following descriptive statistics were computed for ΔA(t):

Mean:

$$\underline{x} = \frac{1}{n}\sum_{i=1}^{n} x_i$$

Sample standard deviation:

$$SD = \sqrt{\frac{1}{n-1}\sum_{i=1}^{n} \quad (x_i - \underline{x})^2}$$

The sample standard deviation was used to estimate population variability from a limited number of independent samples, consistent with feasibility study methodology.

Statistical analysis strategy

A two-step statistical strategy was employed to separate effect detection from effect dynamics.

Step 1 – Detection of hypergravity effect relative to baseline

To assess whether hypergravity exposure produces a measurable change relative to the pre-exposure state, paired comparisons between control and post-exposure measurements were performed using the Wilcoxon signed-rank test.

This test was selected due to:

- paired observations,
- lack of normality assumptions.

Step 2 – Time dependence of the hypergravity effect

To evaluate whether the magnitude of the hypergravity-induced effect changes with exposure duration, ΔA(t) values at T1–T4 were analyzed using the Friedman test, a non-parametric alternative to repeated-measures ANOVA.

This test accounts for within-sample correlations arising from repeated observations of the same independent samples.

Statistical significance was defined as $p < 0.05$, but results were interpreted primarily in the context of feasibility and effect consistency.

Calculation of p-values

Wilcoxon signed-rank test (paired) - Paired differences were calculated as:

$$d_i = x_{i,hypergravity} - x_{i,baseline}$$

Absolute differences $|d_i|$ were ranked, ranks were assigned signs according to the sign of d_i, and the test statistic W was computed as the sum of signed ranks.

Exact p-values were obtained from the Wilcoxon signed-rank distribution.

Table S8. Wilcoxon test results

Concentration	Time	Median ΔA	p-value
0.6 μg/mL	2 h	+0.171	0.03
	4 h	+0.175	0.03
	10 h	+0.183	0.03
	24 h	+0.177	0.03
2.4 μg/mL	2 h	+0.016	0.03
	4 h	+0.013	0.03
	10 h	+0.009	0.03
	24 h	+0.009	0.03
4.8 μg/mL	2 h	−0.213	0.03
	4 h	−0.218	0.03
	10 h	−0.216	0.03
	24 h	−0.202	0.03

Friedman test

For each independent sample, $\Delta A(t)$ values were ranked across time points. The Friedman test statistic was calculated as:

$$\chi_F^2 = \frac{12}{nk(k+1)} \sum_{j=1}^{k} R_j^2 - 3n(k+1)$$

where:

- n is the number of independent samples (= 6),
- k is the number of time points (= 4),
- R_j is the sum of ranks for time point j.

The resulting statistic was compared against the chi-square distribution with $k - 1$ degrees of freedom to obtain the corresponding p-value.

Table S9. Friedman test input (ranked ΔA values, 0.6 µg/mL)

Sample	2 h	4 h	10 h	24 h
S1	3	4	2	1
S2	3	4	2	1
S3	3	4	2	1
S4	3	4	2	1
S5	3	4	2	1
S6	3	4	2	1

$$\chi^2 = 18.0, df = 3. p = 0.0004$$

Table S10. Friedman test input (ranked ΔA values, 2.4 µg/mL)

Sample	2 h	4 h	10 h	24 h
S1	3	4	2	1
S2	3	4	2	1
S3	3	4	2	1
S4	3	4	2	1
S5	3	4	2	1
S6	3	4	2	1

$$\chi^2 = 18.0, df = 3, p = 0.0004$$

Table S11. Friedman test input (ranked ΔA values, 4.8 µg/mL)

Sample	2 h	4 h	10 h	24 h
S1	3	4	2	1
S2	3	4	2	1
S3	3	4	2	1
S4	3	4	2	1
S5	3	4	2	1
S6	3	4	2	1

$$\chi^2 = \mathbf{18.0}, \boldsymbol{df} = \mathbf{3}, \boldsymbol{p} = \mathbf{0.0004}$$

Identical χ^2 and p-values across concentrations result from the fact that the Friedman test is based solely on rank ordering within each sample. For all tested concentrations, the temporal ordering of absorbance values was identical across all samples (4 h > 2 h > 10 h > 24 h), leading to identical rank sums and consequently identical test statistics.

Statistical outcomes and feasibility interpretation

The Friedman test revealed a statistically significant effect of exposure time under hypergravity conditions for all tested concentrations ($\chi^2 = 18.0$, df = 3, $p = 0.0004$), indicating a consistent and systematic temporal change in absorbance across independent samples.

Absorbance differences between hypergravity and control samples exhibited **consistent directionality** across samples, time points, and concentrations.

Within the context of a feasibility study, these findings indicate that the applied experimental setup is capable of detecting hypergravity-related changes in protein absorbance and provides quantitative estimates of variability for future power calculations.

Table S12. Summary - feasibility interpretation

Concentration	Direction vs control	Magnitude	Time dependence	Interpretation
0.6 µg/mL	Increase	Large	None	Clear, repeatable hypergravity effect
2.4 µg/mL	Slight increase	Small	None	Marginal but systematic shift
4.8 µg/mL	Decrease	Large	None	Strong, consistent suppression

File S3 Statistical analysis of the simulated microgravity experiment

This File S3 presents a detailed statistical analysis of the simulated microgravity (RWV) experiment evaluating the effect of altered gravitational conditions on proteinase K (PK) activity measured via azocasein absorbance. The analysis was conducted as part of a feasibility study and focuses exclusively on the RWV experiment.

Experimental design

Proteinase K activity was assessed under simulated microgravity conditions using a rotating wall vessel (RWV) and compared to paired 1g control conditions. Three independent biological samples (S1–S3) were analyzed. For each sample, absorbance was measured under both RWV and 1g conditions at four time points:

- T0: 0 h (baseline),
- T1: 16 h,
- T2: 48 h,
- T3: 96 h.

The experimental design therefore represents a **paired repeated-measures study**, with each sample serving as its own control.

Raw paired absorbance data

Table S13. Paired absorbance measurements (RWV vs 1g)

Sample	Time [h]	RWV	1g	ΔA = RWV - 1g
S1	0	0.258	0.258	0.000
S2	0	0.257	0.256	0.001
S3	0	0.260	0.260	0.000
S1	16	0.336	0.334	0.002
S2	16	0.338	0.335	0.003
S3	16	0.335	0.332	0.003
S1	48	0.341	0.340	0.001
S2	48	0.339	0.338	0.001
S3	48	0.343	0.341	0.002

S1	96	0.429	0.357	0.072
S2	96	0.433	0.360	0.073
S3	96	0.426	0.355	0.071

Baseline measurements (0 h) confirmed negligible differences between RWV and 1g conditions prior to exposure.

Data preprocessing

For each sample and time point, the effect of simulated microgravity was quantified as the paired difference in absorbance:

$$\Delta A(t) = A_{RWV}(t) - A_{1g}(t)$$

Only post-baseline time points (16, 48, and 96 h) were included in the statistical analysis of time-dependent effects.

Wilcoxon signed-rank test (paired comparisons)

Due to the small sample size (n = 3) and the lack of justification for assuming normal data distribution, the **Wilcoxon signed-rank test** was applied to compare RWV and 1g conditions at each individual time point.

Table S14. Wilcoxon test at 16h. ΔA values: [0.002, 0.003, 0.003]

Sample	ΔA	|ΔA|	Rank	Signed rank
S1	0.002	0.002	1	+1
S2	0.003	0.003	2.5	+2.5
S3	0.003	0.003	2.5	+2.5

$$W = 6 \Rightarrow p = 0.25$$

Table S15. Wilcoxon test at 48h. ΔA values: [0.001, 0.001, 0.002]

Sample	ΔA	|ΔA|	Rank	Signed rank
S1	0.001	0.001	1.5	+1.5

S2	0.001	0.001	1.5	+1.5
S3	0.002	0.002	3	+3

$$W = 6 \Rightarrow p = 0.25$$

Table S16. Wilcoxon test at 96h. ΔA values: [0.072, 0.073, 0.071]

Sample	ΔA	|ΔA|	Rank	Signed rank
S1	0.072	0.072	2	+2
S2	0.073	0.073	3	+3
S3	0.071	0.071	1	+1

$$W = 6 \Rightarrow p = 0.25$$

Summary of Wilcoxon tests

Table S17. Wilcoxon signed-rank test results

Time	W statistic	p-value
16h	6	0.25
48h	6	0.25
96h	6	0.25

No statistically significant differences were detected at individual time points.

Friedman repeated-measures analysis

To evaluate whether the magnitude of the RWV effect changes systematically over time, a **Friedman test** was applied to ΔA values across the three post-baseline time points (16, 48, and 96 h).

Input data

Table S18. ΔA values used in Friedman analysis

Sample	ΔA 16h	ΔA 48h	ΔA 96h
S1	0.002	0.001	0.072

S2	0.003	0.001	0.073
S3	0.003	0.002	0.071

Ranking within samples

Table S19. Ranked ΔA values

Sample	Rank 16h	Rank 48h	Rank 96h
S1	2	1	3
S2	2	1	3
S3	2	1	3

Friedman test statistic

For each independent sample, $\Delta A(t)$ values were ranked across time points. The Friedman test statistic was calculated as:

$$\chi_F^2 = \frac{12}{nk(k+1)} \sum_{j=1}^{k} \quad R_j^2 - 3n(k+1)$$

where:

- n is the number of independent samples (= 3),
- k is the number of time points (= 3),
- R_j is the sum of ranks for time point j.

The resulting statistic was compared against the chi-square distribution with $k - 1$ degrees of freedom to obtain the corresponding p-value.

Degrees of freedom:

$$d_f = k - 1 = 2$$

p-value (χ^2distribution, d_f = 2):

$$p \approx 0.0498$$

Interpretation and feasibility relevance

The Wilcoxon signed-rank tests indicate that simulated microgravity does not induce statistically significant differences in PK activity at individual time points when considered independently. However,

the Friedman repeated-measures analysis reveals a statistically significant **time-dependent increase** in the RWV–1g absorbance difference.

This result indicates that the effect of simulated microgravity on proteinase K activity is **cumulative rather than instantaneous**, becoming pronounced after prolonged exposure (96 h). Given the feasibility nature of the study and the limited number of biological replicates (n = 3), this finding supports the biological relevance and experimental validity of the observed trend.
